# Bioinformatics analysis of prognostic value of *PITX1* gene in breast cancer

**DOI:** 10.1042/BSR20202537

**Published:** 2020-09-10

**Authors:** Qiaoyun Wang, Shuai Zhao, Lei Gan, Zhixiang Zhuang

**Affiliations:** 1 Department of Oncology, The Second Affiliated Hospital of Soochow University, No.1055, Sanxiang Road, Gusu District, Soochow 215004, Jiangsu Province, P.R. China; 2 Department of Oncology, Shanghai Fourth People’s Hospital Affiliated to Tongji University School of Medicine, No.1279, Sanmen Road, Hongkou District, Shanghai 200434, P.R. China; 3 Department of Breast and Thyroid Surgery, Wuzhong People’s Hospital of Soochow, No.61 Dongwu North Road, Soochow 215128, Jiangsu Province, P.R. China

**Keywords:** bioinformation, breast cancer, PITX1, prognosis

## Abstract

Background: Paired-like homeodomain transcription factor 1 (*PITX1*) participates in miscellaneous biological processes including cell growth, development, progression and invasion in various malignant tumors. However, the analysis of the association between *PITX1* expression and the survival in breast cancer remains unclear. Methods: Clinical prognostic parameters and survival data related to *PITX1* in breast cancer patients were performed using the bioinformatic analysis including Oncomine, Bc-GenExMiner v4.3, PrognoScan and UCSC Xena. Results: We found that *PITX1* gene expression was significantly higher in different histological classification of breast cancer*.* The Scarff–Bloom–Richardson (SBR) grade, Nottingham prognostic index (NPI), estrogen receptor (ER) negative, epidermal growth factor receptor-2 (HER2) positive, lymph node positive, triple-negative status and basal-like status were positively correlated with *PITX1* level, except for patients’ age and the progesterone receptor (PR) status. We have found that the increased *PITX1* expression correlated with worse relapse-free survival, disease specific survival and overall survival. *PITX1* was positively correlated with metastatic relapse-free survival and distant metastasis-free survival. We also confirmed positive correlation between *PITX1* and the nucleotide-binding oligomerization domain 2 (*NOD2*)*.* Conclusion: The lower expression of *PITX1* was associated with better clinical prognostic parameters and clinical survival in breast cancer according to the bioinformatic analysis.

## Introduction

Breast cancer is the most common malignant tumor in women and the primary cause of female cancer death worldwide [1]. Although the application of locoregional surgery, conventional chemotherapy, precision radiotherapy, endocrine therapy and monoclonal antibody has significant benefits for the prognosis of breast cancer patients, still lots of patients were subjected to the threaten of recurrence and death. Prognosis prediction of breast cancer is related to clinical, pathological and molecular characteristics [2]. Therefore, the identification of new prognostic markers can provide new insight to early detection of breast cancer and decrease the mortality and recurrence.

Paired-like homeodomain transcription factor 1 (*PITX1*) participates in cell growth and development. It was considered as a binary transcription factor involved in the transcription of proopiomelanocortin gene, which may play a role in the differentiation and formation of pituitary cells [3]. In addition, the role of *PITX1* in the development of the hind limbs is mainly found in the areas such as cartilage joints, long bones and skeletal muscles [4]. Haploid deficiency of mice and human *PITX1* can lead to talipes equinovarus [5]. Therefore, *PITX1* was identified as the key role of the hind limb development.

*PITX1* is down-regulated as a tumor suppressor in various malignant tumors and correlated with poor prognosis in gastric carcinoma [6], lung carcinoma [7], head and neck squamous cell carcinoma [8], colorectal carcinoma [9], hepatic carcinoma [10], cutaneous malignant melanoma [11], osteosarcoma [12] and clear cell renal cell carcinoma [13]. *PITX1* promotes the expression of RAS negative regulators as a transcription factor [14]. The tumorigenic effect of *PITX1* gene was achieved by some signaling pathways, such as EMT and WNT/β-catenin signaling pathway that have a positive regulatory effect on proliferation and invasion in gastric cancer [15], while it was related to KRAS, WNT, NF-κB activation pathway and TGF-β signaling pathway in colon cancer [16]. The important role of *PITX1* in the development of malignant tumor can be observed from cancer development, differential expression in cancer and related molecular mechanisms. However, the analysis of *PITX1* in breast cancer is rare and the association between *PITX1* expression and breast cancer patients’ survival remains unknown.

The present study assessed the correlation between *PITX1* expression and the prognostic factor of breast cancer by using various online analysis databases to explore the prognostic significance of *PITX1* gene in the treatment of breast cancer.

## Methods and materials

### Oncomine

Oncomine (http://www.oncomine.org), a cancer microarray database and web-based data mining platform, aims to compare the transcriptome data with normal tissues in most major types of cancer [17]. The gene expression level of *PITX1* was analyzed by Oncomine. We compared mRNA level of *PITX1* for each microarray data between tissue of normal individual and breast cancer patients with designed parameters including 2-fold change, *P* value ≤ 1E-4 and top 10% gene rank. *T-*test was used to analyze the expression difference between different breast cancer pathological types and normal tissues in datasets of *PITX1* gene overexpression. In addition, the co-expression gene of *PITX1* was analyzed concurrently.

### Breast cancer gene-expression miner v4.3 (Bc-GenExMiner v4.3)

Bc-GenExMiner v4.3 (http://bcgenex.centregauducheau.fr/BC-GEM/GEM-Accueil.php?js=1) was analyzed from 36 annotated genomic datasets and three statistical mining functions. Open access database for Bc-GenExMiner v4.3 to analyze the relationship between the expression level of breast cancer specific mRNA level of *PITX1* gene and the specific clinicopathological characteristics of breast cancer (including age, Scarff–Bloom–Richardson (SBR) grade, Nottingham prognostic index (NPI), estrogen receptor (ER), progesterone receptor (PR), epidermal growth factor receptor 2 (HER2), nodal status, triple-negative status and basal-like status). Survival analysis was exerted with the software. Moreover, the relationship between co-expression genes of *PITX1* was analyzed with the database. Data last updated in July 2019.

### PrognoScan

PrognoScan (http://dna00.bio.kyutech.ac.jp/PrognoScan/index.html) is the microarray database of the biological relationship between gene expression and clinical prognosis in various cancers [18]. We utilized PrognoScan database to verify the correlation between mRNA level of *PITX1* expression and survival with the adjusted cox *P* value < 0.05 in breast cancer.

### UCSC xena

UCSC Xena (http://xena.ucsc.edu/) is a genome-related database including many tumor research database functions, providing visual analysis for public data centers. Heat map of co-expression gene can be analyzed in the data mining of The Cancer Genome Atlas (TCGA) by UCSC Xena browser.

## Results

### Overexpression of *PITX1* gene in breast cancer patients

**Figure 1:**
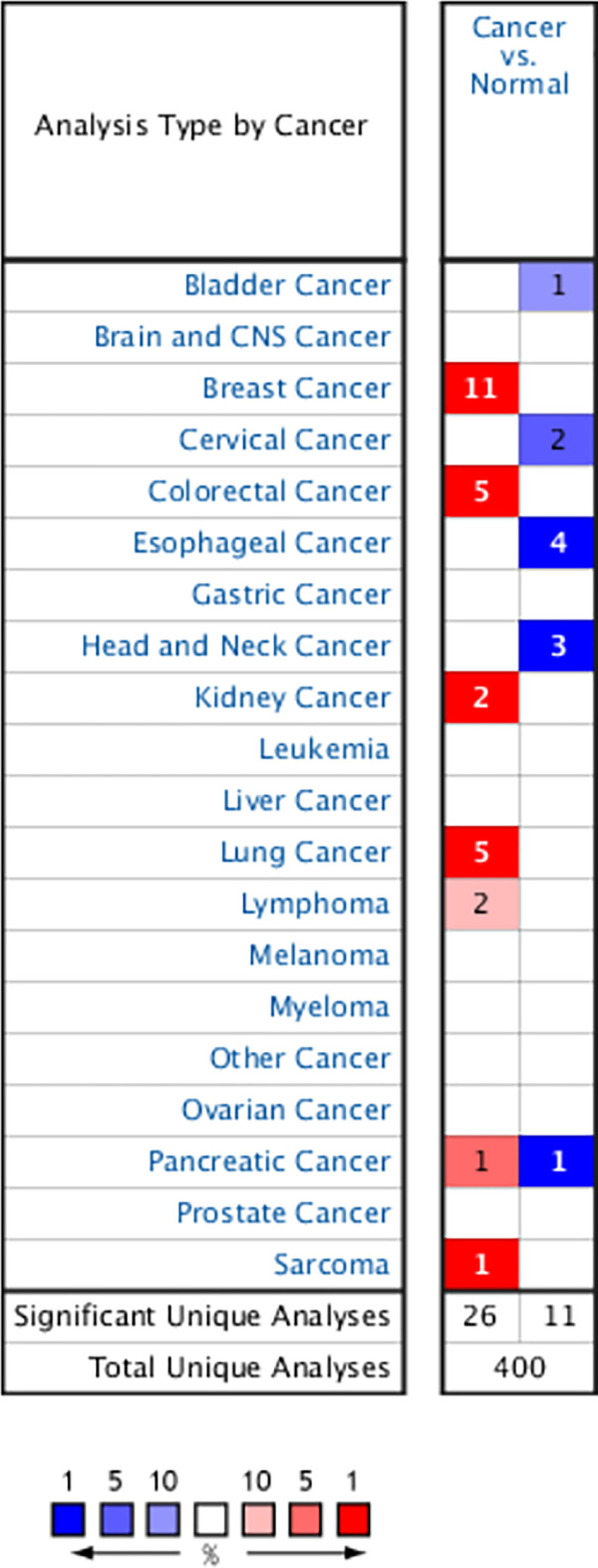
Expression of *PITX1* gene in 20 common tumors compared with paired normal tissues Oncomine database was designed with fold change ≥ 2, *P* value ≤ 1E-4 and gene rank ≥ top 10%. The graphic represents the numbers of datasets with statistically significant (*P*<0.01) mRNA over-expression (red) or down-expression (blue) of *PITX1* (different types of cancer vs. corresponding normal tissue).

**Figure 2:**
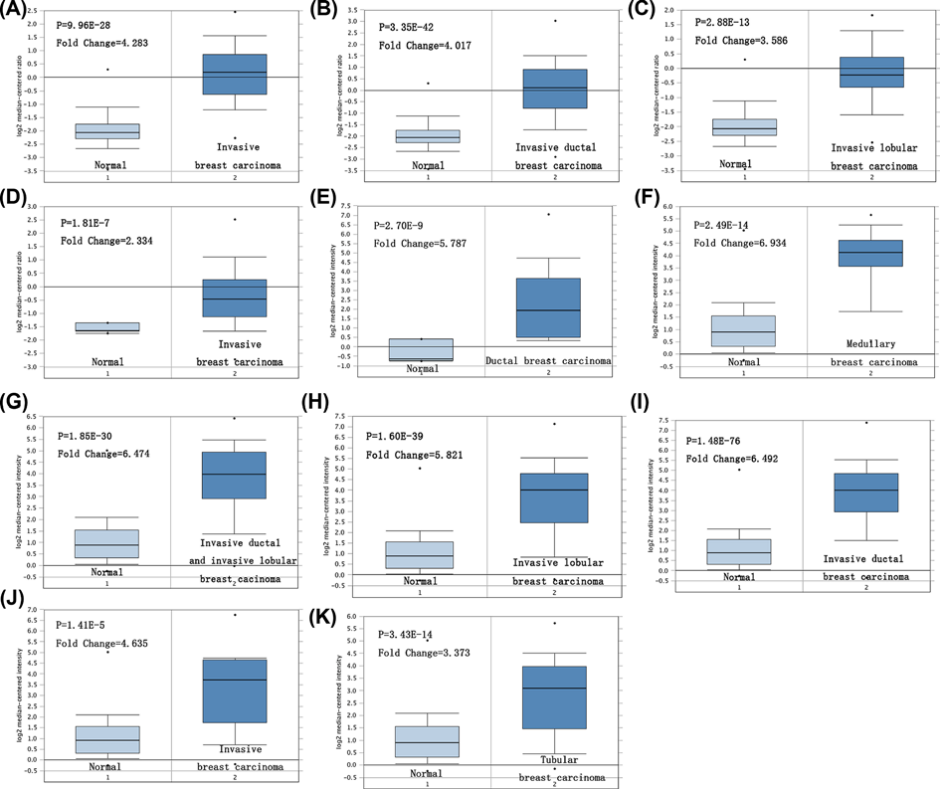
Box plots of normal and tumor differentially expression of *PITX1* gene in different subtypes of breast cancer (**A**) Invasive Breast Carcinoma, (**B**) Invasive Ductal Breast Carcinoma, (**C**) Invasive Lobular Breast Carcinoma, (**D**) Invasive Breast Carcinoma, (**E**) Ductal Breast Carcinoma, (**F**) Medullary Breast Carcinoma, (**G**) Invasive Ductal and Invasive Lobular Breast Carcinoma, (**H**) Invasive Lobular Breast Carcinoma, (**I**) Invasive Ductal Breast Carcinoma, (**J**) Invasive Breast Carcinoma and (**K**) Tubular Breast Carcinoma.

The expression of *PITX1* gene compared with normal individuals and breast cancer patients was detected in 20 kinds of common cancers with tumor online database Oncomine. Up-regulation of *PITX1* gene expression was found in breast cancer, colon cancer, kidney cancer, lung cancer and lymphoma, while down-regulation of *PITX1* gene expression being detected in bladder cancer, cervical cancer, esophageal cancer and head and neck cancer. Four out of ten meet the threshold, which are the 11 datasets of overexpression of *PITX1* gene level from a total number of 43 breast cancer datasets (Figure 1). Compared with normal individuals, the level of *PITX1* gene expression was significantly higher in invasive breast carcinoma, invasive ductal breast carcinoma, invasive lobular breast carcinoma, ductal breast carcinoma, medullary breast carcinoma, invasive ductal and invasive lobular breast carcinoma and tubular breast carcinoma (Figure 2A–K, *P*=9.96E-28, 3.35E-42, 2.88E-13, 1.18E-7, 2.70E-9, 2.49E-1.4, 1.85E-30, 1.60E-39, 1.48E-76, 1.41E-5 and 3.43E-1.4). In the eleven datasets, three datasets of invasive breast carcinoma appears to be the same trend (Figure 2A, *P*=9.96E-28, Figure 2D, 1.18E-7, Figure 2J, 1.41E-5, Table 1), while two datasets of invasive ductal breast carcinoma (Figure 2B, 3.35E-4, Figure 2I, 1.48E-76, Table 1) and two datasets of invasive lobular breast carcinoma are the same (Figure 2C, 2.88E-13, Figure 2H, 1.60E-39, Table 1).

**Table 1:** Different datasets to analyze *PITX1* gene expression in pathological classification of breast cancer

Breast cancer subtype	*P* value	*T* test	Fold change	Sample
Invasive Breast Carcinoma	9.96E-28	14.046	4.283	137
Invasive Ductal Breast Carcinoma	3.35E-42	19.777	4.017	450
Invasive Lobular Breast Carcinoma	2.88E-13	9.583	3.586	97
Invasive Breast Carcinoma	1.81E-7	10.351	2.334	158
Ductal Breast Carcinoma	2.70E-9	7.431	5.787	47
Medullary Breast Carcinoma	2.49E-14	11.369	6.934	176
Invasive Ductal and Invasive Lobular Breast Carcinoma	1.85E-30	14.787	6.474	234
Invasive Lobular Breast Carcinoma	1.60E-39	15.824	5.821	292
Invasive Ductal Breast Carcinoma	1.48E-76	29.168	6.492	1700
Invasive Breast Carcinoma	1.41E-5	5.276	4.635	165
Tubular Breast Carcinoma	3.43E-14	8.774	3.373	211

### *PITX1* gene expression with different clinical parameters in breast cancer

**Figure 3:**
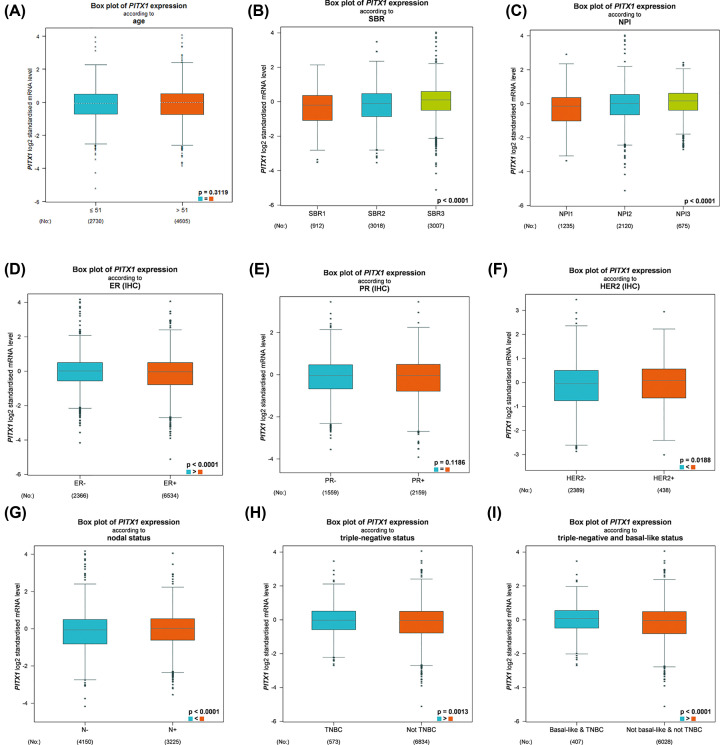
Bc-GenExMiner v4.3 to evaluate *PITX1* gene expression with box plots according to clinical parameters in breast cancer patients (**A**) Age, (**B**) SBR grade, (**C**) NPI, (**D**) ER, (**E**) PR, (**F**) HER-2, (**G**) nodal status, (**H**) triple-negative status and (**I**) basal-like status.

Bc-GenExMiner v4.3 software was used to evaluate the expression of *PITX1* gene with several clinical parameters in breast cancer patients. There was no significant difference of *PITX1* expression between the <51-year-old group and the >51-year-old group (Figure 3A, *P*=0.3119, Table 2). The Scarff–Bloom–Richardson grading system (SBR Grade) is the histological grade which based on tumor size (<2 cm, 2–5 cm, ≥5 cm), lymph node status (positive or negative) and vascular invasion status (positive or negative) in breast cancer [19]. The Nottingham Prognostic Index (NPI) is based on histopathological factors (tumor size, lymph node stage and tumor grade) [20]. The expression of *PITX1* gene was higher with advanced SBR grade and NPI of breast cancer patients (Figure 3B, *P*<0.0001, Figure 3C, *P*<0.0001). The expression of *PITX1* gene was higher in ER negative breast cancer patients (Figure 3D, *P*<0.0001, Table 2). There was no significant difference between the PR positive and PR negative groups (Figure 3E, *P*=0.1186, Table 2). The expression of *PITX1* gene was higher in HER2 positive breast cancer patients (Figure 3F, *P*=0.0188, Table 2). Compared with lymph node negative patients, the expression of *PITX1* gene in lymph node positive patients increased (Figure 3G, *P*<0.0001, Table 2). In addition, *PITX1* was significantly higher in triple negative and basal breast cancer patients than in non-triple negative and non-basal breast cancer patients (Figure 3H, *P*=0.013, Figure 3I, *P*<0.0001, Table 2).

**Table 2:** *PITX1* gene expression analysis in different clinical parameters of breast cancer with Bc-GenExMiner v4.3

Variables	No. of patients	*PITX1* mRNA	*P* value*
Age			0.3119
≤51	2730	–	
>51	4605	–	
**ER**			<0.0001
Negative	2366	Increased	
Positive	6534	–	
**PR**		–	0.1186
Negative	1559	–	
Positive	2159	–	
**HER-2**			0.0188
Negative	2389	–	
Positive	438	Increased	
**Nodal status**			<0.0001
Negative	4150	–	
Positive	3225	Increased	
**Triple-negative status**			0.0013
Non-triple-negative	6834	–	
Triple-negative	573	Increased	
**Basal-like status**			<0.0001
Non-basal-like	7496	–	
Basal-like	1958	Increased	

* Statistical significance was determined by the Welch’s test.

### The effect of the expression of *PITX1* gene on prognosis in breast cancer

**Figure 4:**
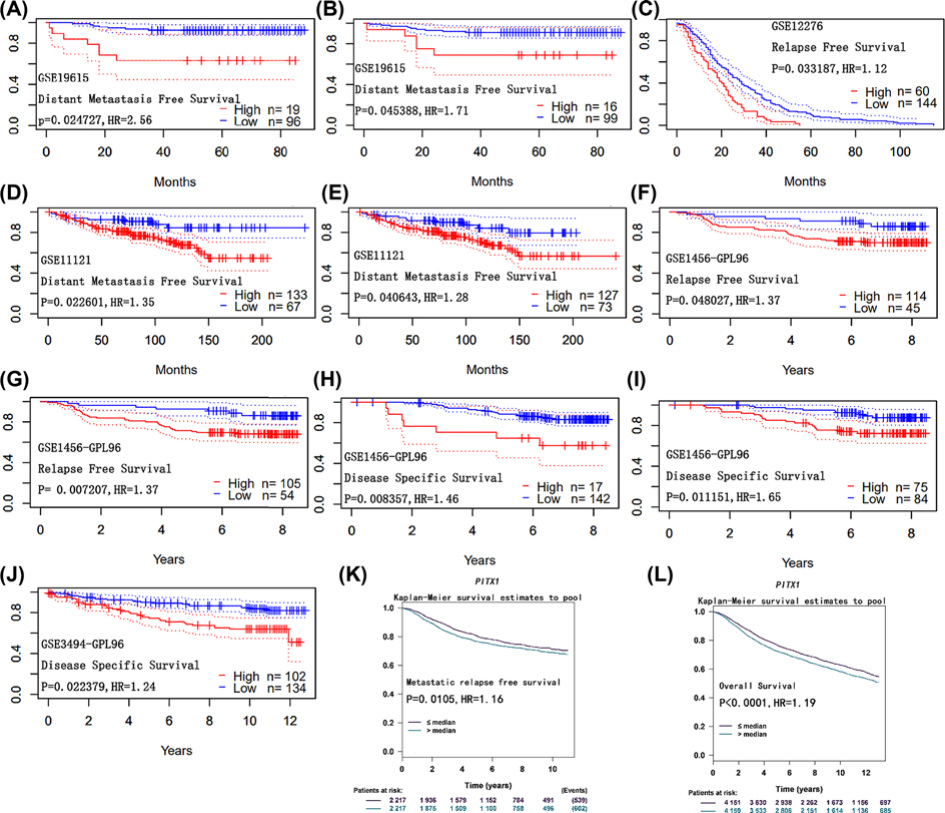
The survival curve of different datasets based on the expression of *PITX1* gene was used to analyze the prognostic value in breast cancer (**A**) Distant Metastasis-Free Survival, (**B**) Distant Metastasis-Free Survival, (**C**) Relapse-Free Survival, (**D**) Distant Metastasis-Free Survival, (**E**) Distant Metastasis-Free Survival, (**F**) Relapse-Free Survival, (**G**) Relapse-Free Survival, (**H**) DiseaseSpecific Survival, (**I**) Disease-Specific Survival, (**J**) Disease-Specific Survival, (**K**) Metastatic relapse-free survival, (**L**) Overall Survival.

The survival curves were plotted with different survival information with survival meta-annalistic software PrognoScan, including distant metastasis-free survival, relapse-free survival and disease-specific survival. Breast cancer patients with *PITX1* (blue) showed positive related with distant metastasis-free survival (Figure 4A, *P*=0.024727, Figure 4B, *P*=0.045388, Figure 4D, *P*=0.022601, Figure 4E, *P*=0.040643, Table 3). The Lower *PITX1* expression group with blue curve was with preferable relapse-free survival (Figure 4C, *P*=0.033187, Figure 4F, *P*=0.048027, Figure 4G, *P*=0.007207, Table 3). The higher *PITX1* gene expression (red) is related with worse disease-specific survival (Figure 4H–J, *P*=0.008357, *P*=0.011151, *P*=0.022379, Table 3). We also analyzed metastatic relapse free survival and overall survival with Bc-GenExMiner v4.3 with the same trend. Expression of *PITX1* (purple) presented positive related with metastatic relapse-free survival (Figure 4K, *P*=0.0105, Table 3) and increased *PITX1* expression presented worse overall survival (Figure 4L, *P*<0.0001, Table 3).

**Table 3:** Different datasets to analyze the prognosis of *PITX1* gene expression in breast cancer

Dataset	Probe ID	End point	No.	Cox *P*-value	HR
GSE19615	Distant Metastasis-Free Survival	209587_at	115	0.024727	2.56 [1.13–5.82]
GSE19615	Distant Metastasis-Free Survival	208502_s_at	115	0.045388	1.71 [1.01–2.89]
GSE12276	Relapse-Free Survival	208502_s_at	204	0.033187	1.12 [1.01–1.24]
GSE11121	Distant Metastasis-Free Survival	209587_at	200	0.022601	1.35 [1.04–1.76]
GSE11121	Distant Metastasis-Free Survival	208502_s_at	200	0.040643	1.28 [1.01–1.62]
GSE1456-GPL96	Relapse-Free Survival	209587_at	159	0.048027	1.37 [1.00–1.88]
GSE1456-GPL96	Relapse-Free Survival	208502_s_at	159	0.007207	1.37 [1.09–1.73]
GSE1456-GPL96	Disease-Specific Survival	208502_s_at	159	0.008357	1.46 [1.10–1.94]
GSE1456-GPL96	Disease-Specific Survival	209587_at	159	0.011151	1.65 [1.12–2.42]
GSE3494-GPL96	Disease-Specific Survival	208502_s_at	236	0.022379	1.24 [1.03–1.49]

### Co-expression analysis of *PITX1* gene

**Figure 5:**
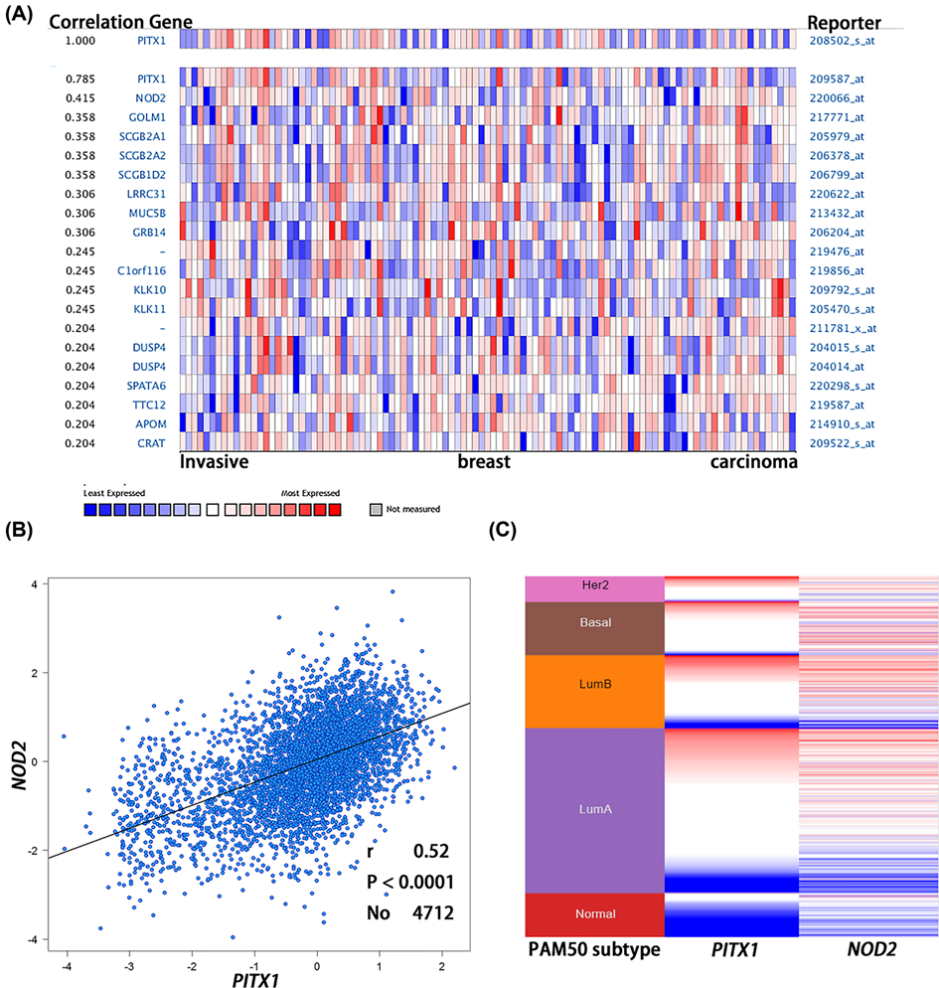
Co-expression analysis of gene *PITX1* and other genes by Oncomine database analysis (**A**) Co-expression analysis of gene *PITX1* in 103 invasive breast cancer with Oncomine database. (**B**). The co-expression gene *NOD2* related to *PITX1* by bc-GenExMiner software (**C**). In TCGA database generated by UCSC Xena tool in breast cancer subtypes, the heat map of *PITX1* and *NOD2* gene expression in 50 gene QPCR analysis (pam50) was analyzed (red represents high expression of gene, blue represents low expression of gene).

Oncomine database was analyzed to assess the related co-expression gene with *PITX1* gene. A total number of 13,363 samples (111 datasets) were searched for the co-expression gene of *PITX1* in Oncomine database. The co-expression profile of *PITX1* was identified with a large cluster of 12,624 measured genes across 103 invasive breast carcinomas, the nucleotide-binding oligomerization domain 2 (*NOD2*) is a principal co-expression gene (Figure 5A). The co-expression gene *NOD2* was associated with *PITX1* positively according to Bc-GenExMiner v4.3 (Figure 5B, *P*<0.0001). We analyzed heat map of *PITX1* and *NOD2* gene expression in 50 gene QPCR analysis (pam50) (Figure 5C) to make sure positive co-expression relationship in breast cancer subtypes of TCGA database generated by UCSC Xena tool.

## Discussion

*PITX1* is a member of *PITX* family member that was considered as development related gene also and tumor suppressor in various carcinoma.

The differential expression of *PITX1* was found in many tumors. *PITX1* binding to the esterogen receptor α (*ERα*) acted on the *ERα* enhancer concentration, and controlled the transcription activity through the binding of *ERα* target gene and *PITX1*-binding site in breast cancer cells [21]. However, there was no prognostic association analysis in breast cancer.

The present study demonstrated that the expression of *PITX1* was up-regulated in breast cancer patients with respect to normal individuals according to Oncomine database. High *PITX1* expression was correlated with poor clinicopathological features in breast cancer. The present results revealed that higher expression of *PITX1* was located in different histological classification of breast cancer with respect to normal individuals, including invasive breast carcinoma, invasive ductal breast carcinoma, invasive lobular breast carcinoma, ductal breast carcinoma, medullary breast carcinoma, invasive ductal and invasive lobular breast carcinoma and tubular breast carcinoma. There was no statistical significance in age and PR status in the analysis. It had been reported that SBR grade and NPI are prognostic factors of breast cancer. The expression of *PITX1* gene was significantly higher in SBR Grade, NPI, ER negative, HER2 positive, lymph node positive, triple-negative and basal-like status, which were related to poor prognosis of breast cancer respectively. Therefore, overexpression of *PITX1* gene may be new biomarker factor related to the poor prognosis in breast cancer. The survival curve of different datasets based on the expression of *PITX1* gene was used to analyze the prognostic value in breast cancer with PrognoScan. According to meta-analysis of survival curve data, ten datasets with clinical statistical value were presented. Breast cancer patients with increased *PITX1* gene showed worse relapse-free survival, disease-specific survival. *PITX1* was positively correlated with distant metastasis-free survival. The same trend also is confirmed that the increased expression of *PITX1* gene showed worse overall survival, and *PITX1* was positively correlated with metastatic relapse free survival in Bc-GenExMiner v4.3.

We also explored the co-expression relationship gene of *PITX1* gene with Oncomine database, which was also verified in Bc-GenExMiner v4.3. *NOD2* were regarded co-expression gene of closely related to *PITX1* genes positively analyzed by Bc-GenExMiner v4.3. *NOD2* increases the risk of breast cancer [22], the research showed that the overexpression of *NOD2* inhibited cell proliferation and promoted clone formation in three negative breast cancer cell lines [23]. The incidence of lymph nodes without metastatic is higher in carriers of NOD2 mutations as a prognostic factor [24]. Therefore, the positive correlation between *NOD2* and *PITX1* gene is reliable.

*PITX1* is down-regulated in various malignant tumors and correlated with poor prognosis, may be because it enrichs in promoters of binding genes and regulates gene expression as a transcription factor [25]. *PITX1* binds to the target gene *PDCD5*, which is an apoptosis related gene, so the down-regulation of *PITX1* is associated with poor prognosis in gastric cancer [26]. DNA hypermethylation silences *PITX1*, and the hypermethylation of *PITX1* is associated with poor prognosis in esophageal squamous cell carcinoma (ESCC) [27]. Hypermethylation of exon 3 of *PITX1* was significantly associated with the risk of death in patients with head and neck squamous cell carcinomas (HNSCC) [28]. There was no prognostic association analysis in breast cancer. High expression of *PITX1* in our research may be because gene methylation level of breast cancer differs from other cancers. As a transcription factor binding ERα in breast cancer [21], high *PITX1* expression may have a certain impact on poor prognosis, which is worth further study.

In general, under the analysis of various bioinformatic tools, the down-regulation of *PITX1* gene was associated with better prognostic clinical parameters such as ER positive, nodal status negative, non-triple-negative, non-basal like status, SBR grade, and NPI. In breast cancer patients, increased *PITX1* expression is correlated with worse relapse-free survival, disease-specific survival and overall survival. *PITX1* was positively correlated with metastatic relapse-free survival and distant metastasis-free survival. It indicates that breast cancer patients may benefit and have better survival with lower expression of *PITX1* gene. Therefore, it may provide some basis for targeted drug therapy related to breast cancer.

## Abbreviations

ER: estrogen receptor
HER2: human epidermal growth factor receptor-2
NOD2: nucleotide-binding oligomerization domain 2
NPI: Nottingham prognostic index
PITX1: paired-like homeodomain transcription factor 1
PR: progesterone receptor
SBR: Scarff–Bloom–Richardson
TCGA: The Cancer Genome Atlas
